# Evaluation of Information Sources in Plastic Surgery Decision-making

**DOI:** 10.7759/cureus.2773

**Published:** 2018-06-09

**Authors:** Nisha Parmeshwar, Chris M Reid, Andrew J Park, Michael G Brandel, Marek K Dobke, Amanda A Gosman

**Affiliations:** 1 School of Medicine, University of California, San Diego, San Diego, USA; 2 Department of Plastic Surgery, University of California, San Diego, San Diego, USA; 3 Department of Neurosurgery, University of California, San Diego, San Diego, USA

**Keywords:** informed consent, internet, patient education, plastic surgery information sources, web-based education, patient satisfaction

## Abstract

Background

Today, patients can access a myriad of information sources regarding plastic surgery procedures prior to meeting with a surgeon. Despite their widespread use, the role of these sources in a patient’s decision-making remains undefined. We hypothesized that the physician remains the key information source for patients making surgical decisions in plastic surgery, but that other sources may deliver important insights and prove helpful to varying degrees. We also explored motivations for this outside information search and any differences in perceived value among patients.

Methods

We administered a survey regarding various information sources to our breast reconstruction, reduction, and abdominoplasty patients. Responses were compared between surgery groups and demographic groups. Ordinal logistic regression analysis was used to determine the impact of patient characteristics on helpfulness rank of different sources.

Results

Survey results were obtained from 58 patients, of whom 10 (17.2%) had abdominoplasty, 35 (60.3%) breast reconstruction, and 13 (22.4%) breast reduction. The most popular information sources prior to the first surgical appointment were Internet searches (56.9%) and family/friends/other patients (39.7%). After the initial appointment, the most useful sources were plastic surgeons (84.5%), and the Internet (36.2%). Most patients (73.5%) still sought outside information after their appointment.

On a Likert-type scale of helpfulness, plastic surgeons ranked 4.28/5, followed by the web-based patient education platform, 3.73 and the Internet, 3.6. A total of 63% of participants listed plastic surgeons as their single most important source of information.

In ordinal logistic regression analysis, non-white race was significantly associated with higher rank of surgeon helpfulness (p < 0.05). Relative to low-income patients, income $50-100k (p < 0.05) and $100k+ (p < 0.05) were associated with lower rank of surgeon helpfulness.

Conclusions

Most patients seek outside information prior to visiting with a surgeon from the Internet, social media, or family and friends. Patients consider plastic surgeons their most valuable information source overall, though still in need of supplementation for varying reasons. Additionally, certain demographic differences affect patient perception of information sources, and this is an important factor for surgeons to consider as they approach educating patients.

## Introduction

Truly informed consent is the result of effective delivery of medical information by a provider, and a patient’s ability to accurately process this information as it guides his decision-making [1, 2]. When achieved, it has strong associations with increased patient satisfaction and decreased anxiety for medical procedures [1, 3].

Plastic surgery, with its diverse range of patients and procedural techniques, may prove especially challenging to obtain a true informed consent. Given that the outcome of procedures may directly affect the quality of life, patients may desire further information, testimonials, or outside endorsements before deciding on surgeon or procedure. Historically, the informed consent process was confined to the physician’s office amidst conversations between doctor and patient. However, in the modern “Internet age,” where information regarding risks, benefits, outcomes, and other facets of medical care is readily accessible, it is unrealistic to assume that patients do not supplement their knowledge base from the outside. Patients are increasingly turning to various Internet platforms to guide their medical decision-making for high-risk procedures [4]. Though physicians may be the top source of information for patients (74%), the Internet is a close second (69%) [5]. As such, some physicians have started to engage in these trends by providing their own links to social media sites [6]. However, according to one survey study of American Society of Plastic Surgery (ASPS) members, only 34% of plastic surgeons found a positive impact of social media on their practice, with the majority reporting minimal use or effect [7].

Likewise, different information sources carry higher levels of influence on different patient populations [8]. Younger patients may be more inclined to search for information online or through social media, placing greater trust in their peers when discussing surgical outcomes and the perceived “worth” of a procedure [9]. As such, a patient’s perception of information sources may influence his surgical priorities and thus may be an important factor for surgeons to address—and qualify—to better serve their patients.

Yet, despite the fact that patients may turn to the Internet for information, it is difficult to assess the efficacy or accuracy of these efforts. One study showed that a preliminary Internet search for “breast cancer surgery” yielded websites that required reading levels far beyond the comprehension level of their patients [10]. The quality of online information requires further scrutiny, given that 67% of websites targeting breast reconstruction are operated by private companies displaying paid advertisements or endorsements [11]. Some argue that these sites do a poor job of contributing to patient decision-making [12], since they are not appropriately vetted or surgeon-driven, but the fact remains: patients are increasingly turning to websites and social media for supplemental medical information.

Literature shows that using a patient-centered approach to informed consent by providing the sufficient and desired information about a surgical experience can meaningfully improve the informed consent process [1, 13, 14]. However, outside information sources play an undefined role in plastic surgery decision-making, and there is still incomplete understanding of their impact on the process. This study aims to elucidate the extent of usage and impact of these sources. We hypothesized that the physician remains the key information source for patients making surgical decisions in plastic surgery, but that other sources including social media may be helpful to varying degrees in delivering important insights and perspectives not otherwise provided by a physician. In addition, we sought to investigate what motivates this outside search for information before and after meeting with the surgeon, as well as any differences in the perceived value of various sources based on individual characteristics.

## Materials and methods

A retrospective survey-based investigation was performed, evaluating the information sources utilized by patients interested in breast reconstruction, breast reduction, or abdominoplasty prior to their surgeries from July to December of 2016. All patients received standard patient education in office as well as an EMMI Solutions® web-based education module specific to the procedure they were considering for a previous quality improvement project performed by our practice [15].

One hundred and twenty-eight patients were emailed a survey that asked which information sources—if any—they used prior to and following their initial consult visit, and how helpful they found each, on a Likert-type Scale. Sources listed included Internet search, social media (support groups, forums, specific pages), family or friends, written pamphlets or books, the EMMI Solutions® educational module, and their surgeon. Additionally, they were asked what they were looking for when they sought information from these outside sources, whether to learn about different techniques of the surgery, understand the risks and benefits of the procedure, set reasonable expectations, acknowledge the recovery time and pain, and/or gather opinions from past patients. Finally, they were asked to identify the single most important source informing their surgical decisions and to rate their overall satisfaction with their decision-making experience.

Available patient demographic information was recorded (Table 1), and comparisons were made between procedure subgroups and demographic subgroups. Ordinal logistic regression analysis was used to determine the impact of patient characteristics on rankings of helpfulness for the various information sources. Covariates included race (white vs. non-white), patient education level (high school degree, college degree, or graduate degree), income ($0-25k, $25-50k, $50-100k, $100k+), and surgery type (abdominoplasty, breast reconstruction, breast reduction).

**Table 1:** Demographic characteristics of survey respondents.

Enrolled Patient Demographics (Percent of reported)	
Race	
White	19 (65.6%)
Black or African American	1 (3.4%)
American Indian or Alaskan Native	1 (3.4%)
Asian	3 (10.3%)
Native Hawaiian or Pacific Islander	0 (0%)
From Multiple Races	1 (3.4%)
Other	4 (13.8%)
Relationship Status	
Married	13 (44.8%)
Widowed	2 (6.9%)
Divorced	8 (27.6%)
Domestic Partnership	2 (6.9%)
Single, Never Married	4 (13.8%)
Annual Income	
$0-$24,999	8 (27.6%)
$25,000-$49,999	7 (24.1%)
$50,000-$74,999	4 (13.8%)
$75,000-$99,999	3 (10.3%)
$100,000-$124,999	1 (3.4%)
$125,000-$149,999	1 (3.4%)
$150,000-$174,999	2 (6.9%)
$175,000-$199,999	2 (6.9%)
$200,000 and up	1 (3.4%)
Education	
No Education	0 (0%)
High School Degree	1 (3.4%)
Some College but No Degree	10 (34.5%)
Associate Degree	2 (6.9%)
Bachelor Degree	6 (20.7%)
Graduate Degree	10 (34.5%)
Working	
No	17 (58.6%)
Yes	12 (41.4%)
Comfort with Technology	
Very Comfortable	19 (65.6%)
Comfortable	7 (24.1%)
Average	1 (3.4%)
Uncomfortable	1 (3.4%)
Very Uncomfortable	1 (3.4%)

## Results

Survey results were obtained from 58 patients, of whom 10 (17.2%) had abdominoplasty, 35 (60.3%) breast reconstruction, and 13 (22.4%) breast reduction. The most popular information sources prior to the first surgical appointment were Internet searches (56.9%) and family/friends/other patients (39.7%). After the initial appointment, the most useful sources were plastic surgeons (84.5%), and the Internet (36.2%). Most patients (73.5%) still sought outside information after their appointment.

**Figure 1:**
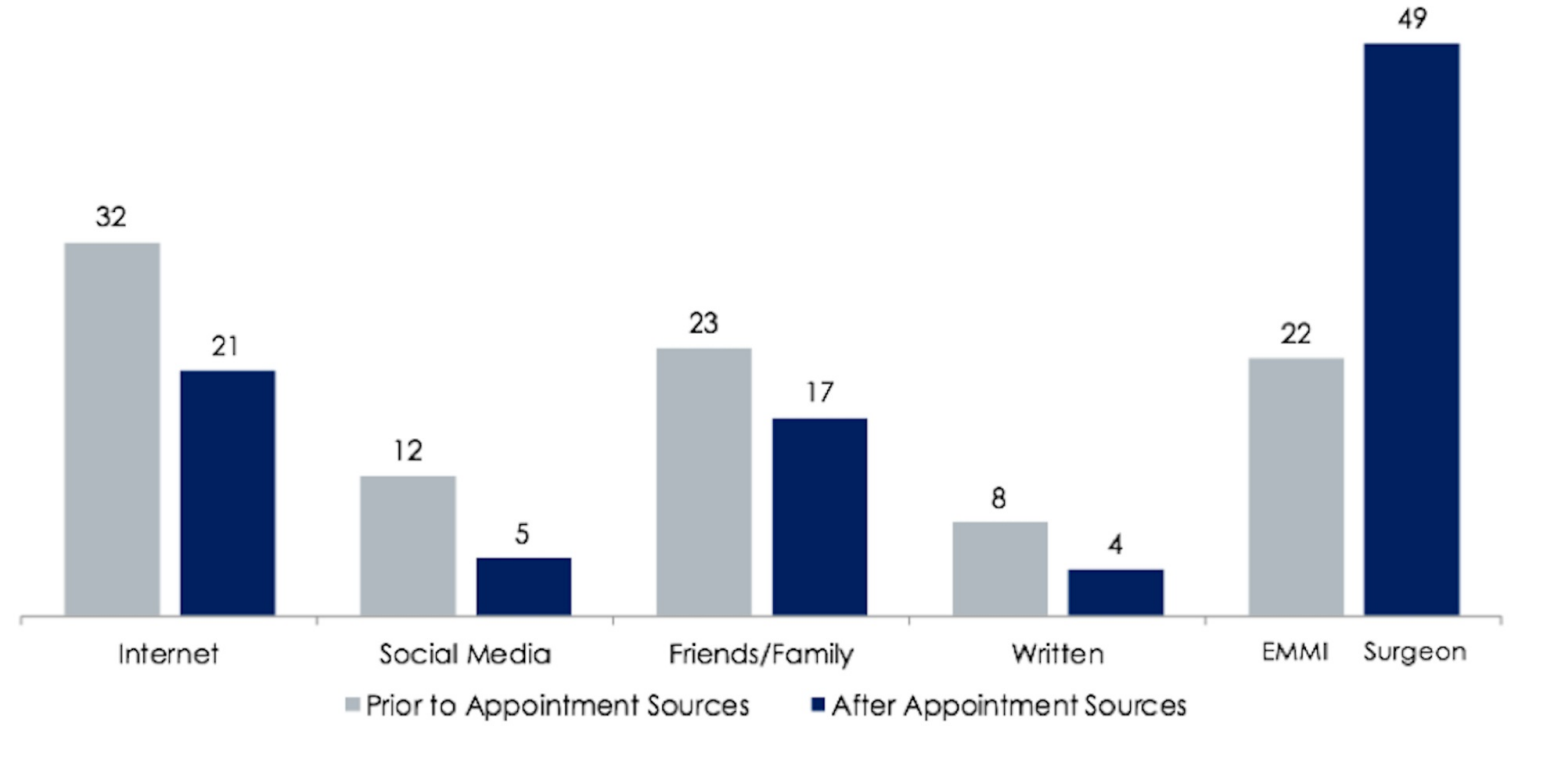
Number of survey respondents who utilized each source prior to their first appointment with the surgeon, and after their initial appointment. Plastic surgery providers are shown as a source after the initial meeting.

Survey results were obtained from 58 patients of whom 10 (17.2%) underwent abdominoplasty, 35 (60.3%) breast reconstruction, and 13 (22.4%) breast reduction within the prior year. The most popular information sources prior to the initial surgical consultation were Internet searches (56.9%) and family/friends/other patients (39.7%) (Figure 1). Nineteen percent of patients did not look for any information beforehand. The top reasons for seeking information before meeting with the surgeon were learning risks and benefits (77.6%), setting reasonable expectations (71.3%), and understanding different techniques of the procedure (67.4%). After the initial meeting with a surgeon, the most utilized sources of information were plastic surgeons and the internet.

**Figure 2:**
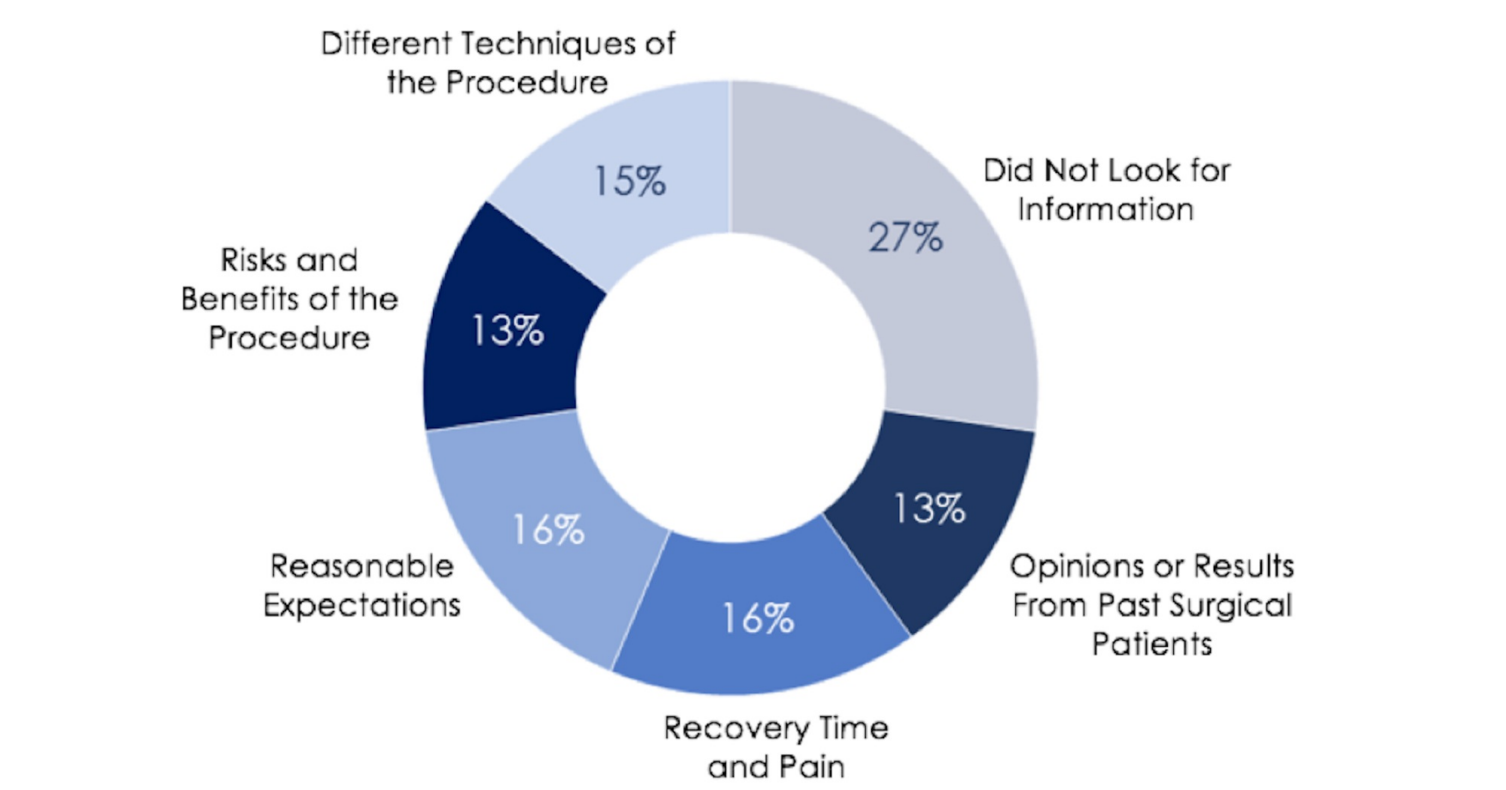
Patient reasons for using outside sources after initial appointment with surgeon.

Interestingly, the majority of people (73.4%) still sought outside information after their appointment, but the reasons for this search were more broadly spread out amongst getting opinions from past surgical patients (13%), setting reasonable expectations (16%), understanding risks and benefits (13%), recovery time and pain (16%), and different techniques of the procedure (15%) (Figure 2). Additionally, nearly half of patients reported asking their family and friends for more information.

**Figure 3:**
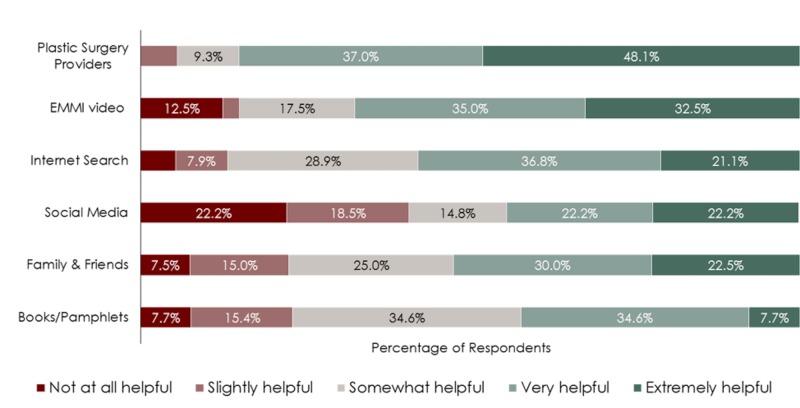
Likert-type rankings of helpfulness for various information sources as rated by survey respondents.

When comparing the various sources on a Likert-type scale of helpfulness, plastic surgeons were ranked as very or extremely helpful by 85.1% of patients, followed by the EMMI educational tool by 67.5% and the Internet at 57.9% (Figure 3). Interestingly social media, though utilized by many patients, was found to be not-at-all helpful or only-slightly helpful by 42.1% of users, and actually had the lowest helpfulness ranking of all sources at 3.0/5. Recalling their surgical experience, 70.3% of participants listed plastic surgeons as their single most important source of information, and overall satisfaction with the surgical experience averaged at 4.15/5.

In ordinal logistic regression analysis, non-white race was significantly associated with higher rank of surgeon helpfulness (p < 0.05). Relative to income below $50k, income $50-100k (p < 0.05) and $100k+ (p < 0.05) were associated with lower rank on surgeon helpfulness. Patient education level and surgery group had no effect. This pattern was the same for the analysis of EMMI helpfulness, demonstrating a positive association with non-white race (p < 0.01) and negative associations with higher levels of income $50-100k (p < 0.05) and $100k+ (p < 0.05).

## Discussion

Informed consent

Despite being a pillar of medical care, informed consent has not been mastered to a level on par with other aspects of surgical care. In the modern information age, the prior dynamic of a surgeon-centered process of informed consent is no longer the norm. Patients are able to initiate the process prior to meeting with a surgeon and with much broader access than in previous decades, increasingly turning to websites and social media for supplemental medical information. Understanding why patients search for outside information and the perceived value of external sources can help surgeons anticipate patient needs, thereby equipping them to achieve true informed consent and increase patient satisfaction. This investigation affirms that patients embark on their learning before meeting with a surgeon and that the majority of patients turn to Internet-based sources for information [5]. Moreover, patients continue to search for supplemental information after their appointment, demonstrating the role of additional outside information as it pertains to informed consent. Nevertheless, the most useful source of information in their surgical decision-making remains the surgeon overall.

Comparison of sources

The ubiquity of the Internet coupled with its unregulated nature creates a quandary for surgeons seeking to accurately inform their patients. This situation may be of particular concern for plastic surgeons, who, compared to other specialties, practice in a more competitive market dependent on referrals from past patients. Nearly all plastic surgeons maintain an Internet site, with online advertising, patient education, and photos being commonplace. In addition, plastic surgeons are increasingly leveraging social media to expand their practice and establish their brand. Our study showed that for our patient population, social media was not helpful in surgical decision making when compared to other sources. However, this may be related to the type of reconstructive or therapeutic procedures included in the study and the academic nature of our practice they were treated in. A similar study performed in a purely private practice aesthetic practice may show drastically different results. Nevertheless, the pervasive nature of social media cannot be ignored. There is a breadth of information regarding plastic surgery procedures online populated by a multitude of sources, all unique in their motivations, biases, and inadequacies. The fact that the majority of patients are turning to the Internet both before and after meeting with a surgeon demands that surgeons manage or participate in this process. This may take the form of attempting to direct patients in online searches or managing their own institution-sponsored online material.

Prior studies have shown that online material falls short of being appropriate, accurate, or approved by surgeons [5, 11, 16]. This may translate to added time during visits to correct issues or concerns that stem from inaccurate information. Our data support that the plastic surgeon remains the most trusted and useful source of information despite the Internet's popularity and accessibility. What is not currently quantifiable and cannot be ascertained from this study is the effect the Internet has on the efficiency of informed consent.

The importance that friends, family, and other patients play in decision making was shown before and after surgical consultation. It is not surprising that patients seek counsel from others close to them that they trust, or those who have undergone the process, which highlights the importance of referrals and maintaining patient satisfaction. The information sources utilized by friends and family were not identified in this study, but they are likely the same as those used by the patient sample.

EMMI® web-based educational tools are designed to supplement physician education of patients. A prior study showed that these tools have no demonstrable impact on the level of informed consent but do serve to bolster patient education provided elsewhere [15]. In this study, EMMI® outperformed the Internet and social media in helpfulness according to patients, likely because they were provided by the surgeon and served as a trusted extension of physician knowledge. The value of these modules in helping surgical decision-making may emphasize the need for other surgeon-recommended or distributed information sources across various platforms to anticipate the needs of our patients outside of the hospital.

Perception of sources and patient motivations

Considering helpfulness of different information sources, it is not surprising that surgeons ranked highest. However, there was a significant discrepancy in affluent white patients valuing the surgeon as a less helpful information source relative to other demographic groups. A similar result was identified for the EMMI® web-based educational tool. One explanation is that this group may have better access to sources of information and therefore may not rely as heavily on the surgeon as the primary information source. Prior studies assert that patients with higher income and education are more likely to go to the Internet for information [4, 17-20]. Interestingly, there was no identifiable difference in how helpful our cohort found different information sources based on education level. This is reassuring that regardless of a patient’s prior education there is uniformity in their appreciation of the available information sources.

Also noteworthy was that social media was perceived as the least helpful source of information by our patient population, despite the fact that many continued to turn to it for guidance. When analyzing the reasons for looking at information, “setting reasonable expectations” and getting “opinions from past patients” became increasingly important to patients after meeting with the surgeon, and “understanding different techniques” and learning “risks and benefits,” became less of key reasons for outside information seeking. This would suggest the technical aspects of surgical informed consent are well delivered to patients by surgeons at their clinic visit. However, there are other important factors weighing into a patient’s surgical decision (i.e., opinions from past patients) which a surgeon alone cannot provide, that the Internet and social media, by means of group communication and forums, can. Surgeons at our institution do not have sponsored social media platforms by which to connect patients to discuss their concerns and experiences, which could have attributed to the low utilization and ranking of social media as a source amongst our patients. However, this does not negate the positive findings of other studies at practices with established social media avenues for patients to get more information [7]. This would suggest that social media is in fact a valid tool for plastic surgeons to utilize in providing the final pieces of data contributing to patient comfort with a surgical decision, which one surgeon alone cannot provide in the office setting. It would be beneficial to explore the impact of surgeon-monitored social media platforms and forums on surgical decision-making, as this study implies there is room and a perceived need for such modalities within plastic surgery practices.

Limitations

This study examines a group of patients who underwent plastic surgery procedures, and provides insights into when and how information is acquired, for what purposes, and differences in perception of various sources. These findings can be helpful to surgeons of all specialties to not only improve patient education but also efficiency in delivering informed consent. However, there are limitations to the study in terms of response rate and lack of certain patient demographics that may affect information sources utilized. Additionally, this study cannot determine how alternative information sources impact the efficiency of patient education as measurable by time spent in clinic visits. For example, a prior report has shown that the EMMI web-based educational platforms actually lengthened plastic surgery clinic appointment times [21]. Anecdotally, we have not found this to be the case. It would thus be of interest to further quantify the effect of different information sources on clinic times. Furthermore, a validated survey may be able to glean finer points of the true impact of sources on informing the surgical decision, and perhaps even estimate level of informed consent given the various information sources. Additionally, reconstructive patients may behave differently from aesthetic patients in regards to outside information seeking, which cannot be gleaned from our survey results. Nevertheless, outside information sources continue to augment informed consent for patients in many surgical specialties and recognizing this fact, and modifying practice accordingly can help to improve patient-centered care across the board.

## Conclusions

A majority of patients seek information prior to visiting with a surgeon, with most searching the Internet or social media, in addition to seeking information from family and friends. As expected, patients consider plastic surgeons their most valuable information source, although they also seek additional information elsewhere and the reasons for utilizing outside sources interestingly change after speaking with their surgeon. This finding demonstrates an important area for surgeons of all specialties to elevate patient-centered care, by directing patients to helpful sources that fill this void, thereby ensuring a multi-dimensional, well-informed surgical decision. Interestingly, there are also demographic differences in how helpful patients consider different sources, and this can be an important insight utilized by surgeons as they approach educating patients for maximum patient satisfaction.
